# Persistent Inflammation and Non-AIDS Comorbidities During ART: Coming of the Age of Monocytes

**DOI:** 10.3389/fimmu.2022.820480

**Published:** 2022-04-11

**Authors:** Ruojing Bai, Zhen Li, Shiyun Lv, Ran Wang, Wei Hua, Hao Wu, Lili Dai

**Affiliations:** ^1^ Beijing Key Laboratory for HIV/AIDS Research, Center for Infectious Diseases, Beijing Youan Hospital, Capital Medical University, Beijing, China; ^2^ Travel Clinic, Center for Infectious Diseases, Beijing Youan Hospital, Capital Medical University, Beijing, China

**Keywords:** monocytes, ART, HIV-1, inflammation, non-AIDS comorbidities

## Abstract

Monocytes are innate immune cells that serve as the first line of defense against pathogens by engulfing and destroying pathogens or by processing and presenting antigens to initiate adaptive immunity and stimulate immunological responses. Monocytes are classified into three types: classical, intermediate, and non-classical monocytes, each of which plays a particular function in response to pathogens. Human immunodeficiency virus type 1 (HIV-1) infection disrupts the balance of monocyte subsets, and the quantity and function of monocytes will not fully recover even with long-term antiretroviral therapy (ART). Monocytes are vital for the establishment and maintenance of HIV-1 latent viral reservoirs and are closely related to immune dysfunction even after ART. Therefore, the present review focuses on the phenotypic function of monocytes and their functions in HIV-1 infection to elucidate their roles in HIV patients.

## Introduction

The monocyte lineage is widely acknowledged to be an essential target cell for human immunodeficiency virus type 1 (HIV-1) infection (1). The balance between monocyte subsets is disrupted after HIV-1 infection, and the number and function of monocytes are not completely restored after long-term antiretroviral therapy (ART) (2), and monocytes can facilitate the HIV-1 reservoir and persistent virus infection (3) as well as affect the initiation/extension of immune activation/senescence and persistent inflammatory events (4). During HIV-1 infection, monocyte subgroups (classical, intermediate, and non-classical) provide additional information about the crucial influence on residual immune dysfunction and increase the risk of non-AIDS-related diseases (cardiovascular disease, neurocognitive disorders, and so on) (5).

ART can significantly inhibit HIV-1 replication while also promoting immune function recovery. With the advancement of “discovery and treatment” strategies, the incidence and mortality of HIV-1 cases are decreasing year by year, making AIDS a controllable chronic disease (6). In the past, our discoveries revealed that monocytes were inhomogeneous with subgroup-specificity phenotypes and roles in the process of HIV-1 infection, and intermediate monocytes were related to illness development in both acute and persistent HIV-1 patients. Anomalies in the proportion of the three monocyte subgroups may be connected to persistent immune activation even after HIV-1 inhibition by ART (2). Herein, our team tracks specific developments in research related to monocyte biological activity and investigates its role, particularly in HIV-1 infection.

## Phenotypic and Functional Subsets of Monocytes

### Monocyte Subset Ontogeny

Over the past 10 years, there has been an increase in knowledge about the biological behavior of monocytes (7). Monocytes were once assumed to have a single phenotype known as uninuclear hematocytes, with a medium dimension between leukomonocytes and granular cells with a kidney-like nucleus. With the development of flow cytometry, monocytes are divided into three types according to the different expression of cellular surface markers bacterial lipopolysaccharide (LPS) receptor CD14 and Fcγ receptor III (FcγRIII) CD16 molecules: classical (CD14^++^CD16^-^), intermediate (CD14^++^CD16^+^), and non-classical (CD14^+^CD16^++^) (8–10).

Multiple lines of evidence support the gradual differentiation of classical to non-classical monocytes in circulation (11). Two studies discovered the enrichment of labeled classical monocytes, intermediate monocytes, and eventually non-classical monocytes in the blood of healthy people using *in vivo* labeling with a brief impulse of 6,6-2H2-glucose (GLU) (12, 13). Similarly, after autologous stem cell transplantation (ASCT), classical monocytes recurred first in the blood after 7 days, followed by intermediate monocytes, and eventually non-classical monocytes after 10 days (14). In addition, posterior to *in vivo* exotoxin treatment in healthy people, monocytes vanished from the blood in 120 min with classical monocytes restoring posterior to 240 min, afterward intermediate monocytes and non-classical monocytes posterior to 24 h (13, 15).

Furthermore, in a separate study using scRNA-seq assay, purified classical monocytes were found to have two subgroups: one with a classical monocyte transcription profile and one with a profile closer to CD16^+^ monocytes, indicating that partial classical monocytes are already on the way to differentiation prior to CD16 upregulation (16). As a result, those results support the hypothesis that classical monocytes are the ancestors of both CD16^+^ monocytes in circulation and monocyte-derived cells within tissue (13, 17).

Moreover, blood monocytes represent 5% to 10% of total white blood cells, and their circulation can persist for 3 days prior to migration into specific locations where they serve as precursor cells to macrophages and (sometimes) dendritic cells (DCs) (18). Additionally, monocytes can migrate with tissues in response to impairment signals, but the clues driving monocyte decision-making on whether to be a monocyte, differentiation, or apoptosis remain unclear (19).

### Monocyte Subsets’ Function

Monocytes are a heterogeneous population with distinct functions of every subgroup in modulating host defenses and inflammation (20). In classical monocytes, the term “classical” is adopted as their phenotype fits the initial depiction of a monocyte (9, 10), comprising 80% to 90% of overall circulating monocytes. Their primary function is to promote phagocytic activity, also known as phagocytic monocyte activity, which is connected with reactive oxygen species (ROS) production (21). Classical monocytes generate cell factors such as TNF and IL-1, as well as a high level of chemokine receptor type 2 (CCR2) expression as homing biomarkers. It is worth noting that classical monocytes are able to convert into CD16^+^ monocytes when stimulated by pathogen-associated molecules (22). The CD16^+^ population is subdivided into intermediate and non-classical monocytes (10, 23).

The features of intermediate monocytes do not necessarily fall midway between classical and non-classical monocytes. Intermediate monocytes comprise 2% to 8% of circulating monocytes (9), and their roles involve ROS generation, antigenic presentation, participation in T-cell proliferation and activation, inflammatory responses, and angiogenesis (9, 20, 23). This subpopulation expresses chemokine receptor type 5 (CCR5) as the primary HIV-1 acceptor, as well as lower levels of CCR2 in contrast to classical monocytes, which induces this type of monocytes to move to the site of infection and invade tissues through CCR2/chemokine ligand 2 (CCL2) (24). Intermediate monocytes are a particular subset of monocytes that are capable of secreting huge amounts of inflammatory cell factors, giving them a strong pro-inflammation capability and a clear role in atherosclerosis in humans (25).

Non-classical monocytes represent 2% to 11% of circulation monocytes (9, 10), and they are naturally mobile and they patrol the endothelial tissue during damage. They express the CX3C chemokine receptor 1 (CX3CR1) at comparatively high levels and are able to protect vascular walls from CX3CR1/chemokine ligand 3 (CCL3) invasive activities when there are stimulating substances (26). Additionally, non-classical monocytes with the Ly6C phenotype are classified as naturally anti-inflammatory by certain researchers since they are essential for vessel endothelial tissue patrol (27).

## Monocytes and HIV-1 Infection

### Monocyte in ART-Treated HIV-1 Infections

HIV infection is considered to begin with a single or small number of transmitted founder viruses at the genital or rectal mucosal surfaces. Monocytes from the circulation are directed to the sites of viral replication and inflammation in the intestines (28). While monocytes are susceptible to HIV infection, their involvement in HIV establishment and spread remains unknown. The establishment of infection in lymphoid tissues, as well as the breakdown of the intestinal barrier, leads to a dramatic increase in plasma viremia, which most likely leads to the infection of perivascular monocytes/macrophages (29). In contrast to other tissue-resident macrophages, perivascular monocyte/macrophages are highly mobile and infiltrate other organs such as the lung and brain. Overall, monocytes and macrophages present many barriers to productive infection, and conditions within these cells are not considered favorable for viral entry and initial replication. However, once the virus surmounts these hurdles and integrates into the host genome, it is likely to persist long-term.

Substantial studies have revealed that HIV-1 infection is related to greater frequencies of CD16^+^ monocytes including intermediate and non-classical monocytes, which are associated with HIV-1 disease progression (30–32). The CD16^+^ monocytes present remarkable susceptibility to HIV-1 infection, as they express comparatively high CCR5 levels (33) and differences in host restriction factor expression (APOBEC isoforms and SAMDH1) (33–38) allowing HIV replication. The level of CD16^+^ monocytes in the overall population is related to non-controlled viremia (>400 copies/ml), decreased by ART treatment, and associated with illness development (31, 32, 39, 40). In addition, studies have recently shown that non-classical and intermediate monocyte expansion is linked to viremia and T-cell activation in elite controllers (ECs) (41, 42). ECs are HIV patients who can regulate their viral loads without the use of antiretroviral therapy (ART), and the majority of them can maintain normal CD4^+^ T-cell counts (43).

Even in settings of effective ART, monocyte disturbance may persist. For example, people with ART treatment exhibited an enlargement of intermediate and non-classical monocytes: comparing participants according to HIV viral load status (60 with “suppressed” HIV RNA levels <400, and 21 with “viremic” HIV RNA levels >400 copies/ml), viremic HIV^+^ patients had significantly higher amounts of intermediate monocytes compared to suppressed HIV (43). The proportions of intermediate monocytes were increased, and the proportions of non-classical monocytes tended to be increased in HIV+ participants (44). In the other HIV/ART group, the frequency of monocyte subsets was similar to controls, but there was immunological dysregulation including both aberrant inflammation and monocyte dysfunction, as well as interindividual variation, implying complex mechanisms connecting monocytes and HIV/ART comorbidities (45).

Moreover, plasmatic soluble CD163 (sCD163) is increased in people with HIV longer than 12 months, and ART treatment decreases sCD163 corresponding to HIV RNA status; nevertheless, sCD163 proportions are still remarkably greater in contrast to the control group, revealing persistent low-level monocyte activation (44). CD163 is a hemoglobin–haptoglobin scavenger acceptor that has only been identified on monocytes and macrophages (45, 46). If monocytes are stimulated by LPS, or FcγR cross-linking or oxidative stress happens, CD163 will be shed as sCD163 to decrease inflammation cell stimulation and cell factor release (44, 47). The expression of CD163 is greater on CD16^+^ monocytes (48). Our lab’s numerical results are consistent with this event. In patients with persistent HIV-1 infection during ART, there was a significant decrease in plasmatic sCD163 proportions and intermediate monocyte surface CD163 density, while surface CD163 levels on intermediate monocytes and sCD163 proportions remained higher than in HIV-uninfected individuals (2). These findings imply that with very long-term ART, there is significant but inadequate normalization of monocyte activation.

### Monocyte in HIV-1 Reservoirs

Monocytes (primarily CD16^+^ monocytes) are also an important component as they are distinct from T cells, which may occur even if ART is available (4). Moreover, circulating monocytes may involve potential viruses and facilitate the expansion of the relevant reservoir following tissue invasion and differentiation into persistent macrophages, forming a long-lived reservoir capable of self-renewal (49). This could happen although the short half-life of monocytes suggests that these cells cannot represent a viral reservoir. However, more research is needed to understand monocytes’ contribution to the macrophage reservoir, such as comparing sequence similarities between macrophage- and monocyte-derived HIV and their differences from T cell-derived sequences from the same individual.

The cause of latent HIV-1 is not fully elucidated in CD4^+^ T cells or monocytes, although it is likely to be a combination of factors such as cytoplasmic sequestration of cell transcriptional factors, epigenesis modulation, and/or transcription inhibitor activity. For example, the positive transcription elongation factor b (P-TEFb), a crucial cell cofactor for Tat, is needed to generate full transcriptions of HIV. P-TEFb comprises a catalysis subgroup, cyclin-dependent kinase 9 (CDK9), and a modulatory subgroup, cyclin T1 (CycT1), which are differently modulated in monocytes in contrast to CD4^+^ T cells (1). Monocytes have quite low levels of CycT1 expression. In comparison, CDK9 expression levels in monocytes remain steady (50). Low levels of CycT1 in monocytes result in poor functional levels of P-TEFb, resulting in low HIV-1 transcriptional outcomes and, as a result, latent virus in monocytes (50).

Latent HIV-1 could be modulated at a posttranscriptional level in monocytes. miRNAs could modulate the expression of host genes at the posttranscriptional level and have also been discovered to affect HIV gene expression (51–53). MiRNA-28, miRNA-150, miRNA-223, and miRNA-382 are discovered to target HIV-1 and cast an effect on the susceptible degree of monocytes to HIV-1. The suppression of those miRNAs in monocytes triggers an elevation of HIV-1 presence (54). These results reveal that miRNAs could be vital for latent virus in monocytes.

Although the HIV-1 infection of monocytes is well designed and relatively well understood, more study is needed to identify the relevant role in the potential virus reservoir. In addition, the developed experiments have to be verified to evaluate the HIV-1 reservoir in monocytes. Due to technological challenges in recognizing the HIV-1 reservoir in monocytes, a further in-depth study is required to define the involvement of infected bone marrow cells in the reservoir, which is also important for the treatment regimen.

### Role in Immune Dysfunction in ART-Treated HIV-1 Infections

Despite that ART recovers CD4^+^ T-cell counts, the abnormal distribution and function of monocytes prolonged and probably facilitated the imperfect restoration of T-cell effector functions. Apart from facilitating a latent reservoir, monocytes cast an effect on immune activation, chronic inflammation, and immune senescence.

#### Promotion of Immune Activation and Chronic Inflammation

Immune activation, as evaluated by the expression of CD38, HLA-DR, or Ki-67 on T cells, has been found to be a better predictor of mortality than viral load in certain studies (55, 56). However, prolonged activation of monocytes may be a more powerful predictor of morbidity and mortality during the virologic suppression process (57). The studies of ECs have provided more evidence for the influence of persistent immune activation on poor outcomes. Nevertheless, persistent immune activation occurs in ECs, which have higher levels of monocyte activation biomarkers such as sCD163, soluble CD14 (sCD14), and CXC chemokine ligand 10 (CXCL10), as well as a higher percentage of CD38^+^HLA-DR^+^CD4^+^ in both EC and chronic HIV-1 compared to HIV-negative controls (58).

In addition, cross-section studies showed that virologically suppressed people with HIV (PWH) display greater levels of monocyte activation (like sCD14 and sCD163) and of circulation plasmatic markers of inflammatory events [like interleukin 6 (IL-6), coagulation cascade activation D-dimer, and C-reactive protein (CRP)] in contrast to age-matched HIV-1-uninfected people (59) but resemble the levels discovered in much older people (60, 61), which coincides with the discoveries from other labs (44, 62).

Furthermore, researchers examined phenotype biomarkers on blood monocytes and discovered the persistent variations in virologically suppressed patients (60). Nevertheless, the plasmatic markers of monocyte activation like CXCL10 are related to monocyte subgroups and phenotype variations (57) and are a better measure for clinic research.

Thus,early in HIV-1 infection activates monocytes, causes systemic inflammation that persists during virologic suppression, and serves as a critical independent predictor of poor prognosis in patients with HIV-1 infection (5). Moreover, there is a potential relationship between cellular innate immune activation and systemic inflammation that may be further improved in the future. Monocyte activation may therefore provide candidate intermediate immunologic end points for future interventional studies targeting persistent inflammation.

#### Promotion of Immune Senescence

Premature immunosenescence occurs in HIV-1 infection, as a result of persistent immune activation and chronic inflammation. Monocytes are vital modulators and effectors in inflammatory events. Inflammation causes a rise in intermediate or non-classical monocytes, which generates massive cell factors and accelerates aging in general (63, 64). Francesco et al. recently discovered a distinct monocyte immune phenotypic profile associated with aging, in which higher levels of CX3CR1, CD64, CD86, and CD91 and lower levels of CD38 and CD40 on diverse monocyte subgroups were the best predictors of aging people including HIV patients, HIV-negative individuals, and blood donors (65). Moreover, monocytes play their roles such as phagocytic function, antigen presentation, and TLR signal transmission, decreasing with aging (63, 66).

The research on HIV-associated immunosenescence has primarily highlighted the acquired immunosystem and discovered biomarkers of acquired immunosenescence such as the enlargement of CD28^-^CD57^+^CD8^+^ T cells and shorter telomeres in CD8^+^ T cells (67, 68) that occur in young HIV patients prematurely (69). Nevertheless, the enlargement of a monocyte subgroup, which expresses CD16 and is discovered to possess a stimulated inflammation phenotype (70) and shorter telomeres (60, 71), is identified in both aged (63, 64, 66) and HIV-infected people (31, 60). Such phenomenon reveals that HIV-associated immunosenescence extends to native immunity as well, especially monocytes. Besides, the discovery reveals that acquired and innate immunity variations are independent of each other in senescence and HIV-1 infection, implying that a parallel but independent causal link may drive innate and acquired immunity variations in the process of HIV infection and explaining the involvement of monocyte immunity parameters, such as CXCL10 and sCD163, as well as acquired immunity parameters, in immunosenescence research (61).

Driving factors of immunosenescence still need more investigation. Despite the fact that ART is related to the amelioration in monocyte immunity disfunction, aging-associated variations to monocyte immunity biomarkers persist even with virology inhibition (60), revealing the participation of other elements apart from HIV-1 viremia. Microbe translocation and consequential endotoxin blood disease, which linger on even with HIV-1 inhibition, are assumed to facilitate prolonged monocyte immunity stimulation and aging; nevertheless, massive other elements such as restimulation of latent viruses might be included as well (72).

### Impact of Monocyte Perturbations on Non-AIDS-Related Comorbidities

It is worth mentioning that the expansion of intermediate and non-classical monocytes has been proposed to have a critical influence on the HIV-mediation pathogenetic process, namely, the initiation and development of cardiovascular diseases and HIV-associated neurocognitive disorders (HAND).

#### Cardiovascular Disease

Monocytes are principal effectors initiating atherosclerosis plaque forming at the locations of endothelium stimulation and impairment (73). The “decision” of monocytes to convert to foamy cells instead of extravasating from the intima affects the initiation and development of atherosclerosis plaque formation (74).

Intermediate monocytes are elevated in HIV-infected patients and serve as an independent predicting factor for cardiovascular diseases, which might be elucidated by completely great levels of pro-inflammation cytokines excreted with the enlargement of this subgroup (75). In comparison to classical and non-classical monocytes, intermediate monocytes demonstrated increased lipidic culmination and oxidized low-density lipoprotein cholesterol (LDL-C) uptake with reduced cholesterol outflow. In addition, intermediate monocytes displayed a comparatively high expression of cholesterol removal acceptors (CD36, CD68) but lowered the expression of the ATP-binding cassette transporter (involved in the outflow of cholesterol) (76).

Non-classical monocytes also exhibit pro-inflammation activity and excrete inflammation cell factors in response to infection, and they participate in antigenic presentation and T-cell activation (77, 78). Non-classical monocytes display great migration ability but merely restricted the phagocytosis effect (78), and these subgroups present varied frequencies in serious coronary artery illnesses (79).

In addition, there is the enlargement of proatherogenic monocyte subgroups (intermediate and non-classical monocytes) with HIV infection of a South African cohort (80). Herein, non-classical monocytes are positively related to immunity stimulation (CD38) and coagulation biomarkers (CD142) expressed on CD4^+^ and CD8^+^ T cells, revealing a correlation with such pathogenic activities in HIV-infected patients. These results unveiled as well a positive association between monocyte subgroups and modulatory T-cell stimulation biomarker glycoprotein A repetitions predominant and specific AT-rich sequence-binding protein 1, revealing both anti- and pro-inflammation effects of monocyte subgroups and suggesting the imbalance of pro- and anti-inflammation status in HIV-infected patients (80). Hence, the effect of every monocyte subgroup on HIV-infected patients is a critical factor, particularly in the background of inflammatory events and cardiovascular disease progression.

Statins have broad anti-inflammatory and immunomodulatory effects, influencing both the innate and adaptive immune responses and affecting a variety of biomarkers of systemic inflammation and endothelial dysfunction that are important in CVD (81). Some studies showed decreases in some or all of the biomarkers studied with atorvastatin and rosuvastatin, mainly T-cell and monocyte activation (82, 83); however, the effect on soluble markers of inflammation has been more inconsistent such as soluble(s) TNF-RII, sCD14, or soluble vascular cell adhesion molecule-1 (sVCAM-1) (84). Further research is needed to determine the significance of these reductions on the outcomes of CVD morbidity and mortality.

Later research should assess the impact of anti-inflammatory drugs, such as those targeting pathogenic substance-induced monocyte stimulation. A better understanding of the transcription variations in monocytes, particularly those involved in cholesterol/lipid metabolic and accumulative activities, will contribute to the discovery of potential target pathways for prevention.

#### HIV-Associated Neurocognitive Disorders

HIV penetrates the central nervous system (CNS) *via* the transmigrating of HIV-positive monocytes, and maybe T cells, across the blood–brain barrier (BBB) (85, 86), which is mediated by elevated chemotactic factors, such as CCL2, in the CNS of PWH despite ART (87, 88). Once in the CNS, monocytes might differentiate into circumvascular macrophages which could form persistent virus reservoirs or release viruses infecting extra CNS cells, such as macrophages, mesoglea, and stellate cells, all of which could be reservoirs even with persistent HIV-1 inhibition *via* ART (89). HIV-positive CNS cells generate host and virus factors, like Tat and Nef, and stimulate more CNS cells, resulting in the release of mediating factors and cell factors with neurotoxicity, causing low-level persistent nerve inflammation and neuron injury (90–94). Such persistent neuroinflammation status lingers on even with ART, mediating the recruitment of extra non-infected and HIV-positive cells, facilitating more CNS virus reservoirs, and probably allowing the duration of HAND.

Late in the infection, monocyte/macrophage traffic induces neuronal damage and maintains viral reservoirs and lesions in the CNS. Williams et al. found that a 3-week injection of the multiple sclerosis drug Tysabri (natalizumab) alleviated CNS damage caused by HIV; more importantly, the drug inhibits infected monocytes/macrophages from infecting CNS through the BBB by inhibiting α4-integrin. CNS is also a reservoir of viruses, and the use of Tysabri in addition to the reservoir activator can further eradicate HIV (95).

Evidence suggests that higher-circulating intermediate monocytes associated with HIV infection are linked to cognitive damage (96–101). Perhaps this is because intermediate monocytes are more likely to transmigrate through the BBB to CCL2 (85). Moreover, HIV-1 intermediate monocytes, matured and infected, are prone to perform the transmigration across the BBB to CCL2 in contrast to HIV^exp^ intermediate monocytes and that such selective benefit is promoted by elevated CCR2, the only known CCL2 acceptor on monocytes, and the junction proteins JAM-A and ALCAM (102). In addition, targeting JAM-A, ALCAM, and CCR2/CCR5 for adjuvant treatment with preexposure prevention or ART methods at present can be imperative for the reduction and/or prevention of generating more CNS virus reservoirs and the treatment of HAND (89).

Additionally, sCD14 and sCD163 are biomarkers that are frequently associated with CNS impairment, specifically cognitive damage (103). Other CNS results, involving psychological health elements like depressive feeling and post-trauma stress, have not been associated with monocyte subgroups within PWH (104). There is evidence in PWH that higher levels of intermediate monocytes can be used to distinguish between people with depressive syndrome and those who do not have those syndromes (105).

Recent studies show that higher levels of intermediate monocytes in the blood are associated with impaired cognition in virologically suppressed HIV women and that they represent a blood-based cognitive marker with accessibility (104). A higher level of overall Toll-like receptor 2 (TLR2)^+^ monocytes in blood is related to improved concurrent cognition, and greater overall TLR2^+^ monocytes are straightly related to the elevation of the classical monocytes cytotype (104). However, more study is needed to find the appropriate cut-point for the proportion of intermediate monocytes predicting cognitive impairment in PWH.

Therefore, it is a fact that the monocytes cast a vital effect on HAND, given that monocytes transmigrate quite preferentially and selectively when monocytes harbor integrated, active viruses (in terms of transcription) and/or with active virus duplication in the ART era in virus reseeding of CNS reservoirs.

## Alternative Treatment Modalities

It was discovered that monocyte-derived macrophages have a high level of dUTP and that this dUTP would be incorporated in HIV DNA following reverse transcription. As these uracil-lated HIV DNA enter the nucleus, the vast majority of uracil-lated HIV DNA are removed by uracil base repair mechanisms (UBER, such as UNG, APE), forcing the virus to undergo abortive infection. Less than 1% of uracil-lated HIV DNA was integrated into host DNA. This partially integrated uracil HIV DNA is unstable and prone to error-prone transcription. Viral DNA isolated from blood monocytes and alveolar macrophages (but not T cells) of drug-suppressed HIV-infected individuals also contained abundant uracils (106). These data imply that this subset of the monocyte–macrophage system is only in interaction with CD4 cells that are reproducing HIV in the local tissue. In addition, these findings also show that using UBER is a unique antiviral strategy for suppressing viral integration; simultaneous inhibition of dUTP may be a target of a brand new integration inhibitor.

Moreover, it is critical to eliminate infected monocytes/macrophages in HIV cure efforts. The study discovered that while NK cells may efficiently kill infected CD4 cells, killing infected monocytes is challenging because interactions with NK cells do not degranulate in response to HIV-infected monocytes (107). HIV CAR-T cells, on the other hand, were discovered to efficiently identify HIV membrane proteins on HIV-infected CD4 and monocyte cells; interestingly, antibodies detect HIV membrane proteins driving ADCC effects of NK cells to kill HIV-infected cells but not on HIV-infected monocytes. Thus, by suppressing NK-cell cleavage, HIV can evade the clearance impact of NK cells on infected monocytes.

In addition, the study found that using a combination of siRNA knockdowns in monocyte-derived macrophages of different TLRs and NLRs as well as chemical inhibition, HIV Vpu could trigger inflammasome activation *via* TLR4/NLRP3 leading to IL-1β/IL-18 secretion (108). The priming signal is triggered by TLR4, but the activation signal is triggered by direct effects on Kv1.3 channels, which result in K+ efflux. In contrast, HIV gp41 could trigger IL-18 production *via* NAIP/NLRC4, independently of priming, as a one-step inflammasome activation. NAIP binds directly to the cytoplasmic tail of HIV envelope protein gp41 and represents the first non-bacterial ligand for the NAIP/NLRC4 inflammasome. These diverse pathways suggest new targets for treating various inflammatory pathologies associated with HIV-1 infection in monocytes and macrophages.

## Concluding Remarks

Monocytes are important components of the congenital immune system, and their subsets play an important role in the HIV pathogenetic process. Monocytes serve as an HIV reservoir throughout the infection process, assisting in the modulation and initiation of immunological activation/senescence and chronic inflammation, and are critical in the initiation and development of non-AIDS-related comorbidities. In light of this, our team suggests that treatment regimens later on ought to target monocytes to selectively regulate abnormal stimulation and inhibit related pathology activities within PWH.

## Author Contributions

RB and ZL wrote the article. SL, RW, and WH edited the manuscript. HW and LD revised the manuscript. All authors contributed to the article and approved the submitted version.

## Funding

This work was supported by the National 13th Five-Year Grand Program on Key Infectious Disease Control (2018ZX10302-102 to ZL), the Beijing Municipal of Science and Technology Major Project (Z211100002921003 to LD), NSFC-NIH Biomedical collaborative research program (81761128001 to HW), National Natural Science Foundation of China (82072294 to ZL), Beijing Natural Science Foundation (7222092 to LD), the Beijing Key Laboratory for HIV/AIDS Research (BZ0089). The funders had no role in study design, data collection and analysis, decision to publish, or preparation of the manuscript.

## Conflict of Interest

The authors declare that the research was conducted in the absence of any commercial or financial relationships that could be construed as a potential conflict of interest.

## Publisher’s Note

All claims expressed in this article are solely those of the authors and do not necessarily represent those of their affiliated organizations, or those of the publisher, the editors and the reviewers. Any product that may be evaluated in this article, or claim that may be made by its manufacturer, is not guaranteed or endorsed by the publisher.

## Abbreviations

HIV-1, human immunodeficiency virus type 1; ART, antiretroviral therapy; LPS, lipopolysaccharide (LPS); FcγRIII, Fcγ receptor III; GLU, glucose; ASCT, autologous stem cell transplant; DCs, dendritic cells; CCR2, chemokine receptor type 2; ROS, reactive oxygen species; CCR5, chemokine receptor type 5; CCL2, chemokine ligand 2; CX3CR1, CX3C chemokine receptor 1; CCL3, chemokine ligand 3; ECs, elite controllers; sCD163, soluble CD163; P-TEFb, positive transcription elongation factor b; CDK9, cyclin-dependent kinase 9; CycT1, cyclin T1; sCD14, soluble CD14; CXCL10, CXC chemokine ligand 10; PWH, people with HIV; IL-6, interleukin 6; CRP, C-reactive protein; LDL-C, low-density lipoprotein cholesterol; sVCAM-1, soluble vascular cell adhesion molecule-1; CNS, central nervous system; BBB, blood–brain barrier; HAND, HIV-associated neurocognitive disorders; TLR2, toll-like receptors2, VL, viral load
